# High temperatures are associated with reduced cognitive performance in wild southern pied babblers

**DOI:** 10.1098/rspb.2023.1077

**Published:** 2023-11-22

**Authors:** Camilla Soravia, Benjamin J. Ashton, Alex Thornton, Amanda R. Ridley

**Affiliations:** ^1^ Centre for Evolutionary Biology, School of Biological Sciences, University of Western Australia, Perth, Western Australia, Australia 6009; ^2^ School of Natural Sciences, Macquarie University, Sydney, New South Wales, Australia 2109; ^3^ Centre for Ecology and Conservation, University of Exeter, Penryn, TR10 9FE, UK; ^4^ FitzPatrick Institute of African Ornithology, University of Cape Town, Cape Town, South Africa, 7701

**Keywords:** behavioural flexibility, inhibitory control, learning, heat stress, climate change, cognition

## Abstract

Global temperatures are increasing rapidly. While considerable research is accumulating regarding the lethal and sublethal effects of heat on wildlife, its potential impact on animal cognition has received limited attention. Here, we tested wild southern pied babblers (*Turdoides bicolor*) on three cognitive tasks (associative learning, reversal learning and inhibitory control) under naturally occurring heat stress and non-heat stress conditions. We determined whether cognitive performance was explained by temperature, heat dissipation behaviours, individual and social attributes, or proxies of motivation. We found that temperature, but not heat dissipation behaviours, predicted variation in associative learning performance. Individuals required on average twice as many trials to learn an association when the maximum temperature during testing exceeded 38°C compared with moderate temperatures. Higher temperatures during testing were also associated with reduced inhibitory control performance, but only in females. By contrast, we found no temperature-related decline in performance in the reversal learning task, albeit individuals reached learning criterion in only 14 reversal learning tests. Our findings provide novel evidence of temperature-mediated cognitive impairment in a wild animal and indicate that its occurrence depends on the cognitive trait examined and individual sex.

## 1. Introduction

Global surface temperature is rising at a rate of 0.2°C per decade and heat extremes are becoming increasingly frequent [1]. The changing climate is affecting the phenology, distribution and behaviour of wild animal populations worldwide [2,3]. Hot extremes can cause lethal heat stroke and dehydration, especially for endotherms already living close to their upper thermal tolerance limits [4]. In Eastern Australia for example, over 23 000 spectacled flying foxes (*Pteropus conspicillatus*) died in a single heatwave in 2018 [5]. Less apparent than mass mortality events but more pervasive are sublethal impacts of high temperatures. A growing number of studies show how behavioural changes adopted in the heat, such as panting, reducing activity or seeking shelter, can result in reduced foraging efficiency, body mass and offspring provisioning [2]. These sublethal effects of high temperatures are predicted to drive future population declines [6,7]. However, one aspect that has been relatively overlooked is how high temperatures may influence animal cognition.

Cognition comprises the mental mechanisms by which an animal acquires, processes, stores and acts on information from the environment [8]. These cognitive mechanisms, including attention, learning, memory and decision-making, can play an important role in recognizing conspecifics, evading predators and remembering the location of resources [9]. Crucially, cognition underpins behavioural flexibility (e.g. [10,11]) and is therefore central to how animals adapt to local conditions [8,12]. Recent research has shown that among 8641 bird species investigated worldwide, those with a greater propensity to use novel foraging behaviours are more likely to show stable or increasing population trends [13]. Additionally, recent studies found that higher cognitive performance can increase survival [14,15]. While such positive relationships have not been found in all species (e.g. [16]), a decline in cognitive performance below the species' average, such as impaired predator recognition, may have detrimental survival consequences [17]. Indeed, even a small cognitive impairment in a single cognitive trait might negatively affect multiple behaviours simultaneously [9,18]. Considering that under an optimistic warming scenario of 1.5°C the frequency of hot extremes is predicted to increase fourfold [1], it is essential to investigate the effects of environmental temperatures on animal cognition. Such knowledge can help to inform conservation and management strategies for vulnerable populations in the face of climate change. Additionally, incorporating temperature thresholds at which cognitive decline occurs to the point of reducing fitness might increase the accuracy of predictions from population viability models under future climate conditions.

To date, most studies investigating the impact of high temperatures on cognitive performance have been conducted in humans [19]. Cognitive impairment in the heat has been associated with worse decision-making and higher injury risk, and is considered an occupational health hazard globally [20]. Additionally, a higher number of hot school days have been related to lower student performance in over 50 countries [21]. In humans, more attention-demanding tasks and tasks that require the individual to resolve conflicting behavioural responses are the most vulnerable to performance decline during heat stress [20]. However, a moderate rise in temperature does not necessarily lead to cognitive impairment [19]. Indeed, faster biochemical reactions in the brain or attentional narrowing can compensate or even overcompensate for heat-mediated cognitive impairment [19]. As temperature increases further, the need to thermoregulate progressively drains attentional resources [22] and cognitive performance declines [19]. This is likely due to a redistribution of cerebral resources among brain regions; it has been suggested that the heating of neurons and temperature-related endogenous feedback act to inhibit output intensity towards cortical areas, which show reduced activity under heat exposure [19]. Similar mechanisms can operate in non-human animals [23–25]. For example, studies in mice (*Mus musculus*) and rats (*Rattus norvegicus*) have shown that brain monoamine levels, which are related to cognitive performance, change during heat exposure [26]. Therefore, non-human animals are expected to suffer similarly from heat-mediated cognitive decline.

Several laboratory experiments have found a heat-mediated decline in animal cognitive performance [18]. For example, at temperatures over 40°C, zebra finches (*Taenopygia guttata*) were unable to discriminate between conspecific and heterospecific calls [27] and to control behavioural responses, i.e. detouring around a barrier instead of pecking it [28]. Similarly, buff-tailed bumblebees (*Bombus terrestris*) took longer to learn the association between a colour cue and a sucrose solution and forgot more quickly at 32°C compared to 25°C, suggesting workers in the wild may be less able to discriminate between flowers and remember the most rewarding ones during hot days [29]. However, most studies investigating heat impacts on cognition have tested animals in captivity [18]; the only study in wild animals found that Western Australian magpies (*Gymnhorina tibicen dorsalis*) were less likely to learn an association between a shape and a food reward when temperatures exceeded 32°C [30]. There is a pressing need for more studies investigating the impact of rising temperatures on cognition in wild animals as there are environmental and behavioural differences (e.g. predation risk and temperature fluctuations) between captive and wild individuals that may affect their learning opportunities and thermoregulatory responses [31,32].

We tested the relationship between heat and cognition in southern pied babblers (*Turdoides bicolor*, hereafter ‘pied babblers’), cooperatively breeding passerines endemic to the semi-arid Kalahari region in southern Africa, which is experiencing rapid warming [7]. Previous research has found that when air temperature exceeds 35.5°C, pied babblers catch significantly less prey during foraging, possibly due to the trade-off between panting and digging in the ground to capture prey [33]. As a consequence, above 38°C their diurnal body mass gain becomes zero [33]. At this temperature threshold, pied babblers' faecal glucocorticoids also start to increase proportionally to air temperature [34]. Additionally, when mean daily maximum temperature exceeds 38°C pied babblers experience a dramatic reduction in fledging success [35]. Population viability models based on these temperature thresholds have estimated that pied babbler populations could experience local extinctions by 2050 [7]. Given the wealth of information on behavioural and physiological impacts of heat in pied babblers, and the potential threat faced under global warming, this species represents an ideal system in which to quantify heat impacts on cognition in the wild.

Here, we measured cognitive performance in three tasks designed to quantify associative learning, reversal learning and inhibitory control in wild pied babblers. These are well-studied cognitive traits that are likely to be ecologically relevant: associative learning and reversal learning measure an animal's ability to learn predictive contingencies between environmental cues and update the learnt associations when conditions change; and inhibitory control measures the ability to inhibit prepotent behavioural responses when counterproductive [36]. In a paired design, we tested each individual in these three cognitive tasks under a non-heat stress condition versus a naturally occurring heat stress condition, which was identified based on the display of heat dissipation behaviours [30]. We predicted that individual cognitive performance in each of these tasks would decline under a heat stress condition. Additionally, since both thermoregulatory responses and cognitive performance have been found to vary with age [37,38], sex [39,40] and dominance rank [41,42], we explored whether these individual attributes interact with temperature to affect cognitive performance. Finally, we also tested whether group size buffers individuals against heat-mediated cognitive decline. Indeed, individuals in larger groups may have a lower heat load at a given environmental temperature due to lower contributions to helping behaviour (load-lightening [43]), such as less time spent incubating or flying to provision offspring [44,45], potentially resulting in reduced cognitive impairment in the heat.

## 2. Methods

### (a) Study site and population

Data were collected at the Kuruman River Reserve (KRR; 26°58′ E, 21°49′ S) in the Northern Cape, South Africa during the austral summers (September–March) of 2018, 2019, 2021 and 2022 (fieldwork was suspended in 2020 due to the COVID-19 outbreak). The KRR is situated in the semi-arid Kalahari region, where the main vegetation is a xeric savanna. The average annual summer precipitation at the KRR is 174.0 ± 70.1 mm and the average summer maximum temperature is 34.5 ± 1.4°C (2005–2020, [46]).

Pied babblers are medium-sized passerines (60–90 g) that live in stable social groups. Groups comprise a dominant pair that produce approximately 95% of the young, and subordinate helpers [47]. Older individuals in the population are on average heavier (Pearson's *r* = 0.47 in the present study) and more likely to be dominant [48]. The dominant pair is identified from aggressive displays towards subordinates [49] and overnight incubation by the dominant female [49]. Each group defends a territory of 50–80 hectares year-round [50]. Group size ranged from three to six adults (i.e. individuals ≥1-year post-hatching) during the study. The study population has been monitored since 2003 and is habituated to human presence [49], which allows direct presentation of cognitive tasks to the birds. Birds in the population are coloured-ringed for individual identification. Ringing is performed on nestlings when they are 11 days old, and on adult immigrants. During ringing, a blood sample is taken for molecular sexing as pied babblers are sexually monomorphic [49]. Therefore, age and sex are known for most individuals in the population. Adult immigrants are assumed to be 1 year old at the time of immigration or two years old if they breed on the first year they immigrate because dispersal and first breeding are rarely recorded before these ages [48,51]. During the breeding season (austral summer), researchers visit each study group weekly and record any breeding activity (nest-building, incubation, offspring provisioning, post-fledging care).

### (b) Cognitive testing

Cognitive performance was quantified using three tasks targeting associative learning, reversal learning and inhibitory control. The cognitive tasks were designed to test abilities that are closely related to the natural terrestrial foraging behaviour of pied babblers [49], by requiring them to search a well on the ground or detour around a barrier to retrieve a mealworm (*Tenebrio molitor* larva). Cognitive testing was always carried out in the shade as pied babblers usually forage under vegetation cover [49] and microclimate variables can vary widely between the sunlit and shaded locations [2]. Prior to cognitive testing, ambient-temperature water was provided to the focal bird to limit potential confounding effects of dehydration on cognitive performance [52]. However, wild animals often experience dehydration and heat stress simultaneously during heatwaves; therefore, our data represent a conservative estimate of the potential impact of heat on cognitive performance in wild animals [18].

To limit the potential influence of social learning on cognitive performance, cognitive testing was performed when the individual was temporarily out of sight from other group members. This was achieved by situating the cognitive task behind a shrub or tree that separated the individual from the rest of the group, or by waiting until the individual moved away from other group members during foraging. This was possible because pied babblers often forage 10 m apart from each other [53] and each trial in a cognitive test takes <1 min, so test subjects had time to complete a trial before another individual approached.

#### (i) Heat condition

**Figure 1.**
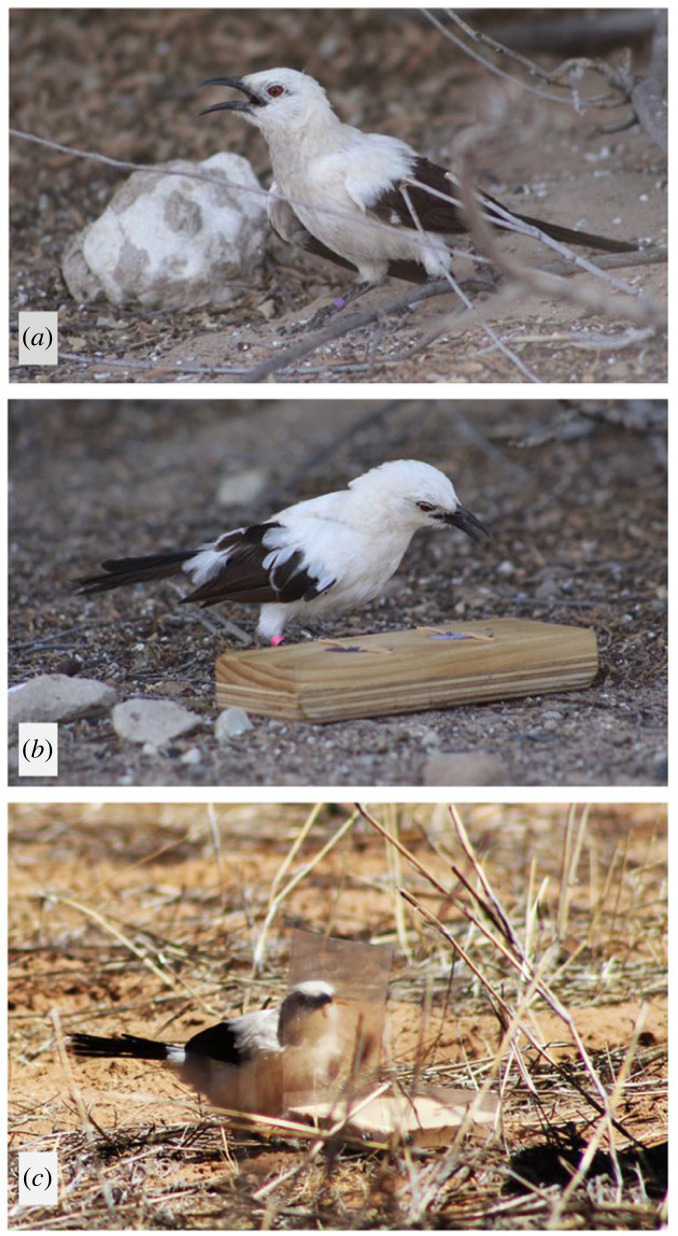
A wild pied babbler (*a*) displaying heat dissipation behaviours (panting and wingspreading); (*b*) interacting with the cognitive task used to quantify associative and reversal learning while panting; (*c*) successfully detouring around the inhibitory control task (from the left side of the transparent wall in this case) and retrieving the mealworm. Photo credits: Nicholas Pattinson.

In a paired design, the same individuals were tested on each task (i.e. associative learning, reversal learning, inhibitory control) in both heat stress and non-heat stress conditions, so that measures of cognitive performance in a given task were balanced across individuals and conditions. The order of the two conditions was randomized for each task. Testing was considered to be under a heat stress condition if the focal bird displayed heat dissipation behaviours (i.e. panting and wingspreading, figure 1*a*) for at least 25% of the testing time. We chose this threshold because (i) pied babblers displayed heat dissipation behaviours for at least 25% of the testing time when the maximum temperature during testing was 35.4°C or above, which aligns with the critical temperature threshold for net 24 h body mass loss in this species [33]; and (ii) 25% was the threshold used in a previous study on heat stress and cognition in wild Western Australian magpies [30], making future comparisons across species possible. Additionally, to avoid ambiguous heat conditions, if the bird stopped displaying heat dissipation behaviours under a heat stress condition or started displaying heat dissipation behaviours under a non-heat stress condition for more than three consecutive trials, the test was paused and continued the following day under the same condition.

At the start of each cognitive test, the experimenter noted down the weather conditions (clear or overcast) and relative humidity, obtained from an on-site weather station (Vantage Pro2, Davis Instruments, Hayward, USA; factory calibration with accuracy = 0.3°C), and measured ground and air temperature with a digital thermometer (RS Pro Rs42 Digital Thermometer, K type input, resolution of 0.1°C, accuracy of ± (0.5% rdg + 1°C)) in the shade within 1.5 m of the focal bird. Air and ground temperature measurements were repeated every 30 min during testing. Cognitive testing took place between 5.30 and 19.00, when pied babblers are active. The heat stress and non-heat stress tests for each task were administered on average 23 ± 2 s.e. days apart to avoid confounding effects of seasonality on cognitive performance. Owing to logistical constraints, we were not always able to perform cognitive testing in both heat stress and non-heat stress conditions at the same time of the day. However, temperatures in the Kalahari can exceed 30°C by 10 am during summer [33], and by including time of day as a candidate explanatory term in the statistical analyses, we were able to assess the relative importance of temperature and time of day for variation in cognitive performance (see §2d).

During cognitive testing, if the bird did not approach the task for 30 min, the test was paused and continued the following day under the same condition (mean number of days per test per individual: 1.3, range 1–4 days). When an individual took more than 1 day of testing to reach passing criterion, the environmental conditions and time of day associated with the cognitive test were recorded as follows: time of day was recorded at the start of the cognitive test on the first day of testing. Humidity was measured at the start of cognitive testing during each testing day and then averaged across days. Weather condition (clear versus overcast) was recorded as ‘overcast’ if the sky was not clear during at least one of the testing sessions. Maximum temperature during testing (*T*_max_) was defined as the highest temperature recorded across testing sessions; this was highly correlated with the average temperature across sessions (Persons' *r* = 0.97, *p* < 0.001).

#### (ii) Associative and reversal learning

The cognitive task used to quantify associative and reversal learning consisted of a wooden block (180 × 70 × 30 mm) with two circular wells (30 mm diameter, 20 mm depth), covered by wooden lids (figure 1*b*). The lids fitted snugly into the wells and were held in place by elastic bands, which allowed them to swivel when pecked, making the food reward accessible. Prior to the associative and reversal learning tests, birds were trained to peck the lids to retrieve a mealworm (see electronic supplementary material, §1). For the associative and reversal learning tests, the task was presented with the two lids painted different shades of a given colour. The food reward was randomly assigned to one of the two colour shades. The use of shades of the same colour instead of different colours limited potential effects of colour preference on performance [54]. Following the protocol of Shaw *et al*. [55] and Ashton *et al*. [36], in the first presentation of the task (i.e. first trial), the bird was allowed to search both wells; in subsequent trials, the bird was only permitted to eat the mealworm if it first pecked the lid of the rewarded colour, otherwise the task was removed. This ensured there was a cost associated with an incorrect choice. There was an inter-trial interval of approximately 1 min before the task was presented again (average inter-trial interval 1.7 min ± 0.1 s.e.). The orientation of the associative learning task was pseudorandomized between trials to avoid the bird forming an association with the position instead of the colour of the lid. A trial was recorded as correct if the bird pecked the rewarded lid. The bird was deemed to have learned the association after six consecutive correct trials (significant deviation from a random binomial probability, binomial test *p* = 0.016). Associative learning performance was quantified as the number of trials until learning criterion. For birds that reached criterion, a reversal learning test was performed 24 h after, following the same protocol but rewarding the previously unrewarded colour shade. If the bird did not reach criterion by 120 trials, the test was stopped and was not included in the analysis of individual cognitive performance.

#### (iii) Inhibitory control

The inhibitory control task consisted of a transparent PVC barrier (clear smooth PVC, 200 µm thick), fixed to a wooden base on the ground (figure 1*c*). A mealworm was positioned behind the barrier and the bird had to inhibit the prepotent behavioural response of pecking the transparent barrier and instead detour around it to retrieve the mealworm. Except for the first trial, if the bird pecked the barrier, the experimenter removed the task before the bird could reach the mealworm. A trial was recorded as correct if the bird detoured around the barrier and retrieved the mealworm without pecking it. A bird passed the inhibitory control task when it performed six correct trials in a row, and inhibitory control performance was quantified as the number of trials to criterion. We did not include an initial training phase, in which the individual is rewarded when it detours around an opaque barrier, because performance in the testing phase could then be influenced by the individual's memory of a learned rule (peck behind the barrier) more than its inhibitory control [36]. However, by allowing individuals to retrieve the mealworm even if they touched the transparent barrier during the first trial, we ensured that all individuals were aware of the presence of a barrier. All birds reached criterion before the cut-off of 120 trials.

#### (iv) Task variants

Since each bird had to be tested on both a heat stress and non-heat stress condition, we used causally identical but visually distinct variants of each cognitive task in the two conditions to avoid the confounding effect of memory on task performance [36]. This meant changing the colour of the lids in the associative and reversal learning tasks, and the shape of the transparent barrier in the inhibitory control task between conditions (see electronic supplementary material, §2). The variants of each task assigned to each bird were randomized within year and condition.

### (c) Proxies of motivation

Food availability in the environment, individual condition and temperature itself can influence the motivation of an individual to interact with food-based cognitive tasks and impact measures of cognitive performance [54]. For this reason, we quantified several proxies of motivation under both heat conditions: relative body mass, foraging efficiency, foraging effort, latency to approach the task and inter-trial interval.

The birds in the study population have been trained to jump on a top-pan scale to access a food reward (mealworm or egg yolk crumbs), allowing researchers to measure their body mass (accuracy ± 0.1 g) weekly during summer [49]. As a proxy of motivation, we used ‘relative body mass’, calculated as the ratio of individual body mass measured within 4 h prior to cognitive testing over the season average (range 0.91–1.11, indicating individuals 9% lighter to 11% heavier than their season average when tested). Additionally, during weekly visits to the groups, we carried out 20-min focal behavioural observations of the birds tested. This entailed recording all behaviours of the focal bird to the nearest second, including foraging and size and number of prey caught, using a custom-designed program in the free software Cybertracker [56]. These data were then used to compute foraging effort (i.e. proportion of observation time spent foraging) and foraging efficiency (i.e. biomass ingested per foraging minute), where the latter was calculated only for focal observations in which the bird spent at least 5 min foraging [57]. Prey items that were provisioned to young were excluded from this calculation to better approximate individual hunger level. We examined the potential effect of foraging effort and efficiency on cognitive performance in the subset of tests for which we could pair heat condition and timing with a focal behavioural observation. This was achieved by: (i) categorizing a focal behavioural observation as under heat stress only if the bird spent at least 25% of the time showing heat dissipation behaviours; (ii) matching cognitive tests and behavioural focals only if they were both performed in the early morning (before 9.00) or later in the day (after 9.00); and (iii) considering only behavioural focals that were carried out within 3 days of the corresponding cognitive test. Finally, we quantified average inter-trial interval and latency to approach the task for each test under both conditions (see electronic supplementary material, §3).

### (d) Statistical analyses

To determine whether heat significantly affected associative learning and inhibitory control performance, we fitted two separate sets of Generalized Linear Mixed Models (GLMMs), with number of trials to pass as the dependent variable and candidate explanatory terms as predictors. The GLMMs were fitted by maximum likelihood (ML), with a negative binomial error distribution to control for overdispersion. We set both group identity and individual identity as random terms in models of inhibitory control performance, and only individual identity in models of associative learning performance because the addition of group identity resulted in model convergence issues and individual identity explained a higher proportion of variance (electronic supplementary material, table S3). To determine whether heat significantly affected reversal learning performance, we fitted a set of negative binomial Generalized Linear Models (GLMs) with number of trials to pass as the dependent variable. We did not include individual ID and group ID as random terms due to the limited sample size of reversal learning tests in which individuals reached criterion (*N* = 14 tests) and because they explained less than 0.001% of variance in performance.

Candidate models were ranked based on their AICc values (Akaike's information criterion corrected for small sample sizes). We removed maximum ground temperature from the candidate terms because it was highly correlated with maximum air temperature (Pearson's *r* = 0.98, *p* < 0.001). When two predictors were moderately correlated (e.g. maximum air temperature and time of day, Pearson's *r* = 0.74, *p* < 0.001), the term with the lowest AICc was included in additive models with other candidate terms. Models within two ΔAICc of the best model and with 95% confidence intervals not intersecting zero were included in the top model set and were considered to explain variation in cognitive performance better than other candidate models [58].

The candidate explanatory terms tested were: age, sex, rank (subordinate versus dominant), weather (clear versus overcast), heat condition (hot: heat dissipation behaviours observed for at least 25% of the testing time, or cold: less than 25%), time of day, maximum air temperature during testing (°C), relative humidity at the start of testing (%), year, ordinal date (calculated as number of days from September 1) and breeding stage (three categories: none, nest, fledglings). We tested group size as a categorical variable (small: three adults and large: at least four adults) because groups of five or six adults comprised only the 13% of the dataset. We also tested motivation proxies: latency to approach (s), inter-trial interval (min), foraging efficiency (g min^–1^), foraging effort and relative body mass. Additionally, we included ‘testing order’ within a group and ‘condition order’ (1 or 2, indicating which test was performed first in each pair of non-heat stress versus heat stress tests) to test for unintended social learning and for performance improvement over successive replicates, respectively. Finally, we examined the potential effects of colour shade (dark versus light) or barrier shape (umbrella, arch, wall, corner, cylinder) on task performance. In models of associative learning and inhibitory control performance, we tested all pairwise interactions of maximum air temperature during testing (hereafter, *T*_max_) with individual and group attributes as well as condition order. We did not test interactions in models of reversal learning performance due to the limited sample size.

Analyses were performed in R version 4.2.0 [59]. All continuous predictors were scaled (centred on the mean and divided by 1 s.d.). GLMMs were fitted with the *glmmTMB* package [60] and we used the *DHARMa* package to simulate model residuals (function ‘simulateResiduals’) and then test them for presence of outliers, dispersion and uniformity (function ‘testResiduals’) [61]. This allowed us to confirm that model assumptions were not violated.

## 3. Results

### (a) Associative learning performance

In 13% of the associative learning tests individuals did not reach criterion, and these tests were excluded from further analysis (three heat stress and three non-heat stress tests out of 46 tests; see electronic supplementary material, table S1). Twenty-one individuals from nine groups (mean group size 3.8 ± 0.9 standard deviation, hereafter ‘s.d.’, range three to six adults) reached criterion in at least one heat condition, for a total of 40 associative learning tests (see electronic supplementary material, tables S1 and S2). In these tests, maximum temperature during testing ranged from 18.0 to 33.0°C (mean 26.4 ± 4.8°C, *N* = 20 tests) under non-heat stress conditions and from 37.0 to 41.2°C (mean 38.4 ± 1.3°C s.d., *N* = 20 tests) under heat stress conditions. For the 17 individuals that reached criterion in the associative learning task under both heat conditions, there was on average an 11.7°C (± 4.7 s.d.) difference in maximum temperature between conditions. Individuals took on average 44.9 trials (± 36.2 s.d.) to reach criterion in the associative learning task under a non-heat stress condition and 56.3 trials (± 34.0 s.d.) under a heat stress condition.

**Figure 2.**
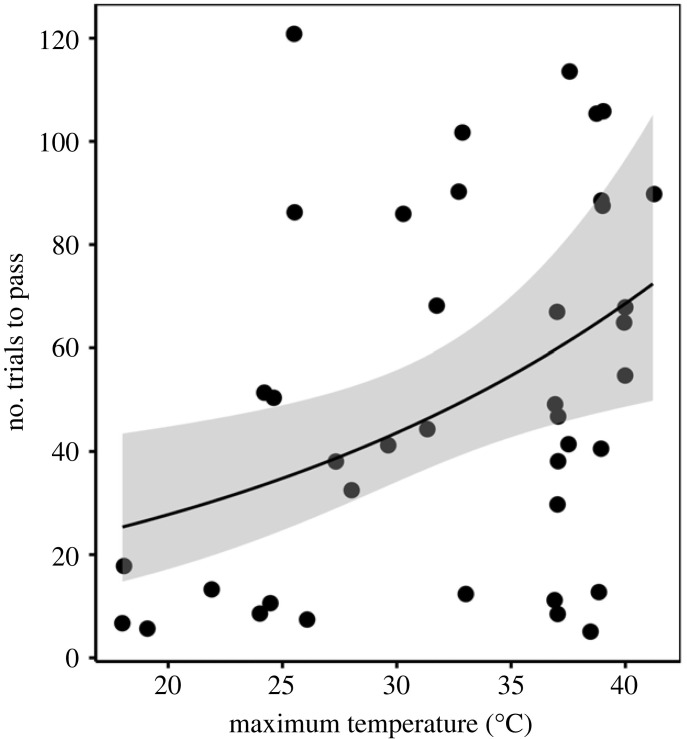
Number of trials to pass the associative learning task as a function of maximum temperature during testing in 21 pied babblers (*N* = 40 tests). Points are raw data and they were slightly jittered on the *x*-axis to avoid overlap. The negative binomial regression line is represented by the solid line and the 95% confidence intervals are represented by the shaded area.

We found that associative learning performance varied significantly with maximum temperature during testing (table 1 and figure 2) but not with heat condition (i.e. ‘*T*_max_’ was a better predictor than ‘heat condition’; see electronic supplementary material, table S4). This indicates that individuals performed worse with increasing temperatures independently of whether behavioural markers of heat dissipation were observed. On average, individuals took twice as long to learn an association between a colour cue and a food reward when temperature during testing reached 38°C compared to 23°C (negative binomial regression equation: ln(number of trials) = 2.419 + 0.045 (*T*_max_), *Z*_40_ = 2.42, s.e. = 0.02; 31 trials at 22.6°C versus 62 trials at 38.0°C).

**Table 1.** Top model sets of candidate terms affecting cognitive performance in three tasks (associative learning: *N* = 40 tests, 21 individuals, 9 groups; reversal learning: *N* = 14 tests, 10 individuals, 6 groups; inhibitory control: *N* = 60 tests, 27 individuals, 9 groups) in pied babblers. For each task, corrected Akaike Information Criterion (AICc) and *Δ*AICc are provided for the top model set. Coefficient estimates ± standard errors (s.e.) and 95% confidence intervals (CI) are given below the top model sets for models that are at least 2 *Δ*AICc from the intercept-only model. See electronic supplementary material, tables S4 (associative learning), S7 (reversal learning) and S8 (inhibitory control) for full model selection outputs.

model selection	AICc	*Δ*AICc
associative learning		
*T*_max_	393.51	0.00
** **intercept-only	396.34	2.83
reversal learning		
colour shade	139.84	0.00
intercept-only	141.19	1.34
inhibitory control		
** ***T*_max_ × sex + relative body mass	498.59	0.00
intercept-only	516.72	18.13
effect sizes	estimate ± s.e.	95% CI
associative learning		
*T*_max_	0.32 ± 0.13	0.06; 0.57
inhibitory control		
*T*_max_	0.36 ± 0.09	0.20; 0.53
sex (males)	0.09 ± 0.13	−0.17; 0.34
relative body mass	−0.34 ± 0.08	−0.49; −0.19
*T*_max_ × sex (males)	−0.50 ± 0.13	−0.76; −0.24

In 23% of the associative learning tests, individuals took more than 1 day to reach criterion. It is important to note that the individuals that took more trials to learn the association were more likely to require multiple days of testing. When controlling for the presence of a pause in cognitive testing (testing days: single = 1, multiple = 0) we still found that individuals required significantly more trials to learn an association as maximum temperature during testing increased (see electronic supplementary material, table S5).

### (b) Reversal learning performance

In 42% of the reversal learning tests (10 tests out of 24) individuals did not reach criterion, independently of maximum temperature during testing and heat condition (see electronic supplementary material, tables S1 and S6). These tests were therefore excluded from further analysis. Ten individuals from six groups (mean group size 3.6 ± 0.8 s.d., range three to six adults) reached criterion under at least one heat condition, for a total of 14 reversal learning tests (see electronic supplementary material, tables S1 and S2). In these tests, the number of trials required to reach reversal learning criterion was not significantly higher than the number required to learn the initial association (Wilcoxon signed-rank test, *V* = 30, *p* = 0.173, *N* = 14).

Individuals took an average of 53.4 trials (± 35.8 s.d.) to reach reversal learning criterion in the non-heat stress condition (*N* = 8 tests) and 60.7 trials (± 27.1 s.d.) in the heat stress condition (*N* = 6 tests). Maximum temperature during testing ranged from 20.0 to 27.0°C (mean 23.5 ± 2.9 s.d.) under non-heat stress and from 35.4 to 41.2°C (mean 38.1 ± 2.3 s.d.) under heat stress conditions. For individuals that reached criterion under both heat conditions (*N* = 8 tests from 4 individuals), there was on average a difference of 14.8°C (± 3.5 s.d.) in maximum temperature between conditions.

Reversal learning performance did not vary significantly based on whether the individual was consistently displaying heat dissipation behaviours or on maximum temperature during testing (table 1; see also electronic supplementary material, table S7). None of the candidate explanatory terms tested were significant predictors of reversal learning performance (electronic supplementary material, table S7).

### (c) Inhibitory control performance

All of the 27 individuals tested (from nine groups, mean group size 3.8 ± 0.8 s.d., range three to six adults) reached criterion in the inhibitory control task under both heat conditions, out of which three individuals were tested twice over the course of the study, for a total of 60 inhibitory control tests (see electronic supplementary material, tables S1 and S2). Maximum temperature during testing ranged from 21.6 to 34.7°C (mean 29.2 ± 3.4 s.d., *N* = 30 tests) in the non-heat stress condition and from 35.7 to 40.3°C (mean 37.7 ± 1.3 s.d., *N* = 30 tests) in the heat stress condition. The difference in maximum temperature between inhibitory control tests under heat stress and non-heat stress conditions for a given individual was on average 9.3°C (± 4.2 s.d.). Individuals completed the inhibitory control task in 29.6 trials (± 19.2 s.d.) and 30.2 trials (± 19.7 s.d.) in the non-heat stress and heat stress conditions, respectively. Pied babblers took significantly fewer trials to reach criterion in the inhibitory control task compared to the associative (Wilcoxon signed-rank test, *V* = 342.5, *p* = 0.024, *N* = 30) and reversal (Wilcoxon signed-rank test, *V* = 61, *p* = 0.014, *N* = 12) learning tasks performed under the same condition.

**Figure 3.**
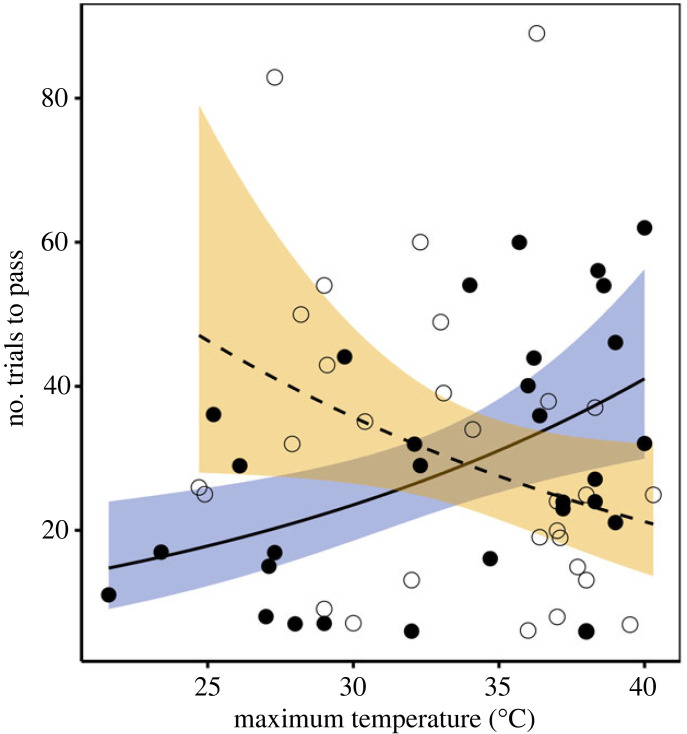
Number of trials to pass the inhibitory control task as a function of maximum temperature during testing and sex (females: filled dots, purple shade, solid line, *N* = 30 tests from 15 individuals; males: empty dots, yellow shade, dashed line, *N* = 30 tests from 12 individuals) in pied babblers. Points are raw data and they were slightly jittered on the *x*-axis to avoid overlap; the solid and dashed lines are negative binomial regression lines and the 95% confidence intervals are represented by the shaded areas.

Heat condition was not a significant predictor of inhibitory control performance (electronic supplementary material, table S8). However, after controlling for the effect of relative body mass (individuals performed better when heavier than their season average; see electronic supplementary material §6), inhibitory control performance depended on individual sex and maximum temperature (table 1). Females, but not males, required more trials to pass the inhibitory control task as maximum temperature during testing increased (females: coefficient ± s.e. = 0.36 ± 0.09, 95% CI = 0.19; 0.53, *N* = 30; males: coefficient ± s.e. = −0.14 ± 0.11, 95% CI = −0.34; 0.07; *N* = 30; figure 3). This sex difference may be partly due to males already performing worse than females under a non-heat stress condition (number of trials in males (reference level = females): coefficient ± s.e. = 0.50 ± 0.17, 95% CI = 0.17; 0.83, *N* = 30 tests). Finally, in 13% of the inhibitory control tests, individuals required more than one day to reach criterion. As for associative learning performance, if we accounted for the gap in cognitive testing experienced by these individuals (testing days: single = 1, multiple = 0) we still found that females required significantly more trials to pass the inhibitory control task as maximum temperature increased (see electronic supplementary material, table S9).

## 4. Discussion

The impacts of heat on cognition may have important implications for animals' ability to adjust their behaviour under global climate change and increasing heatwave frequency. Here, we tested wild pied babblers on associative learning, reversal learning and inhibitory control tasks under naturally occurring heat stress and non-heat stress conditions. We found clear evidence for temperature-mediated cognitive decline in associative learning performance, whereby wild pied babblers took more trials to learn the association between a colour cue and a food reward as maximum temperature during testing increased from 18°C to 42.3°C. This temperature-mediated decline in associative learning performance aligns with evidence from captive studies in mice [62], rats [63] and buff-tailed bumblebees [29], and recent evidence from wild Western Australian magpies [30].

Pied babblers required on average twice as many trials to learn an association at 38°C compared to moderate temperatures (23°C). Above 38°C, pied babblers also experience a rise in faecal glucocorticoid metabolite levels [34], which may represent a physiological mechanism behind the observed temperature-mediated learning impairment. Indeed, in humans and other mammals, plasma cortisol concentration increases following heat exposure [23,64], and some studies have found that high cortisol levels are associated with reduced cognitive performance [64,65]. Importantly, neither time of day nor motivation proxies explained variation in associative learning performance, suggesting that lower motivation or reduced activity in the heat were not the main factors underpinning the observed temperature-mediated learning impairment. Additionally, the fact that individuals completed up to 120 trials in the heat indicates that they were still able to search the wells, ruling out impaired motor function as an explanation for the decline in cognitive performance.

In the inhibitory control task, we found that females required more trials to consistently refrain from pecking the transparent barrier as temperature increased. This finding provides the first empirical evidence for a temperature-mediated decline in inhibitory control in a wild animal, and it aligns with the findings of a previous study examining inhibitory control performance in captive zebra finches [28]. However, in pied babblers, only females showed a temperature-mediated performance decline. Given that pied babblers’ faecal glucocorticoids increase linearly with air temperature above 38°C [34] and can vary according to rank and sex [66], it is possible that glucocorticoid levels increase differently with temperature in male and female pied babblers, potentially explaining the observed sex difference in inhibitory control decline [67].

Alternatively, the observed sex-specific cognitive impairment could be due to inherent sex differences in inhibitory control, which are present in numerous animal taxa [40]. Accordingly, in pied babblers, males showed worse inhibitory control than females under a non-heat stress condition. Considering that breeding competition negatively impacts female fitness [68], selection may favour better inhibitory control in females, at least under moderate temperatures, in order to successfully navigate competitive interactions. For example, better inhibitory control could allow females to inhibit the instinct of mating when dominant females are present [69] or interacting aggressively with other females when the chances of winning a fight are low [70].

In both the associative learning and inhibitory control tasks, individual performance was predicted by temperature but not heat condition (defined via heat dissipation behaviours). This may be partly due to the variability in temperature within heat conditions, allowing a continuous measurement of temperature to explain statistically more variance in cognitive performance. However, it also suggests that at moderately high temperatures some individuals may exhibit heat dissipation behaviours and not experience cognitive decline, while at very high temperatures some individuals might show cognitive impairments despite not continuously showing such behaviours. The former group may be able to efficiently use heat dissipation behaviours to maintain thermal homeostasis and prevent temperature-mediated cognitive decline, at least under a moderate temperature increase [5,18]. The latter group may use facultative hyperthermia, instead of adopting heat dissipation behaviours, to reduce water loss [5]. The increase in body temperature above normal may explain the decline in cognitive performance for these individuals [18]. Individuals may also shift between thermoregulatory strategies depending on their internal state (e.g. recent food intake and breeding activities) and environmental conditions (e.g. rate of temperature change during the day, current position within their territory). Therefore, relying solely on heat dissipation behaviours may not be sufficient to identify the onset of cognitive decline, and temperature should always be measured alongside behavioural observations.

We also found that, despite the occurrence of load-lightening in pied babblers [43], group size did not buffer individuals against temperature-mediated cognitive decline. Indeed, there was no interactive effect of group size and temperature on cognitive performance in associative learning or inhibitory control tasks. This aligns with a previous study that found no evidence for differences in general cognitive performance between pied babblers in different-sized groups [38]. These findings however should be treated with caution as they are based on a limited range of group sizes (nine different social groups, ranging in size from three to six adults).

Performance in different cognitive tasks showed a different sensitivity to increasing temperatures, with associative learning declining across all individuals, inhibitory control declining only in females and reversal learning performance showing no temperature-related variation. Notably, in a given heat condition, pied babblers required significantly fewer trials to achieve criterion in the inhibitory control task compared to associative and reversal learning tasks, suggesting that inhibitory control may be less cognitively demanding. Given that in humans less cognitively demanding tasks often do not show a temperature-related performance decline [20], this could explain the weaker effect of temperature on inhibitory control compared with associative learning performance. The same does not apply to the reversal learning task: the fact that individuals failed to reverse the previously learnt association in 42% of the tests, reaching learning criterion in only 14 tests, suggests that this task was cognitively demanding. Despite this, we found no decline in reversal learning performance at high temperatures. While it might be premature to fully exclude temperature effects on reversal learning given the limited sample size, it is theoretically possible that impaired associative learning—and possibly impaired memory [38]—in the heat actually favours the reversal process by reducing proactive interference. Proactive interference occurs when a previously learnt association interferes with new learning by causing repeated choices of the now unrewarded cue [71]. Hence, impaired associative learning in the heat might lead individuals to ‘forget’ previously learnt associations more quickly, which could compensate for slow learning of the new (reversed) association and lead to the absence of measurable differences in reversal learning performance between temperatures.

Ultimately, our findings suggest that as temperatures rise, wild animals will likely become less able to learn associations between environmental cues and they may show less behavioural inhibition. This, in turn, could lead to poorer decision-making during foraging, social interactions and predator defence [72–74]. The implications of temperature-mediated cognitive impairment are concerning as it may limit animals' ability to adaptively adjust behaviour when facing habitat disturbance, accelerating population declines [13]. Future studies linking individual measures of cognitive performance to behavioural responses, such as discrimination of alarm calls [75] or predator recognition [76], under a range of temperatures will be a critical next step to assess to what extent temperature-mediated cognitive decline is associated with impaired behavioural decisions. This might be particularly important for cognition-based conservation [77]. For example, some reintroduction projects are training captive-release animals to associate predator models with danger [78,79], or invasive toxic prey with sickness [80], which have helped improve survival rates in the wild [78,80]. Therefore, knowing how increasing temperatures might affect animal cognition, such as the ability to learn an association, might improve conservation outcomes in the face of climate change [18].

## Data Availability

Supplementary material is available online [81].
